# Development of a Patient and Carer Advisory Board to Co‐Design Health Services Research for the Quality of Care of People With Dementia

**DOI:** 10.1111/hex.70662

**Published:** 2026-04-05

**Authors:** Daniel X. Bailey, Jane Thompson, Elizabeth Beattie, Paul Prudon, Leanne Jack, Jenni Lawson, Karyn Lendich, Elizabeth Miller, Glenys Petrie, John Quinn, Ivy Webb, Leonard C. Gray, Melinda Martin‐Khan

**Affiliations:** ^1^ Australian Centre for Health Services Innovation (AusHSI) and Centre for Healthcare Transformation, School of Public Health & Social Work Queensland University of Technology (QUT) Brisbane Queensland Australia; ^2^ Centre for Health Services Research, Faculty of Health Medicine and Behavioural Sciences The University of Queensland (UQ) Brisbane Queensland Australia; ^3^ Member of the Evaluating Quality Care (eQC) Patient and Carer Advisory Board; ^4^ School of Nursing Queensland University of Technology Brisbane Queensland Australia; ^5^ School of Nursing, Midwifery & Social Sciences Central Queensland University Brisbane Australia; ^6^ Department of Health and Community Services, Exeter Medical School University of Exeter Exeter UK

**Keywords:** codesign, consumer and community involvement, dementia, lived experience expert, public and patient involvement

## Abstract

**Introduction:**

Patient and Public Involvement (PPI) is becoming increasingly common in research and is now a requirement by many funding bodies. In this paper, we describe a PPI Board that was formed to bring lived experience expertise to the research conception, development and implementation of the evaluating quality care (eQC) project, which focussed on improving the care of older people and people living with dementia in acute care hospitals.

**Methods:**

We discuss how the board was initially conceived and developed, including the process to recruit an appropriately skilled and experienced Chair and members, and operational aspects of the co‐design focused Board.

**Results:**

The Board's membership, conduct of meetings and outcomes are described. The Board's eight members provided advice and input on research ideas and protocols. They co‐authored 16 publications and provided advice and input on a further 12 publications for three PhD students linked with the project.

**Discussion:**

Important aspects of PPI, including successes and challenges of setting up a PPI Board are discussed as well as recommendations for other groups who are interested in developing a PPI Board as co‐design partners in research.

**Patient and Public Contribution:**

The eQC public and patient involvement board comprises people living with dementia and carers of people living with dementia. This article was conceived of and jointly planned with the eQC PPI Board and eQC research team. The research team members wrote the first draft (D.B. and P.P.), and the Board edited the manuscript. All Board members are listed as authors.

## 1 Introduction

The Patient and Carer Advisory Board (the Board hereon) was established to support the Australian National Health and Medical Research Council (NHMRC) funded evaluating quality care (eQC) project. The eQC project was funded for 3 years from 2019 but was extended to 2024 due to pandemic‐related delays and focused on improving the quality of hospital care for older people, particularly those living with dementia and cognitive impairment. It is estimated that 55 million people are living with dementia worldwide and it is one of the major causes of disability, costing the global economy approximately USD 1.3 trillion annually [1]. In Australia, the proportion of the population over 65 is conservatively projected to reach 21% by 2066, up from 16% in 2020 [2]. Consequently, the demographics of hospital admissions will likely shift to reflect this. It is well established that older patients, especially those with dementia or other cognitive impairments are at greater risk of adverse events in acute care [3, 4]. Therefore, innovation of care delivery and management of conditions associated with age are research priorities. To effectively address these challenges, it is essential to include the perspectives of patients, carers, families and communities [5, 6]. This inclusion produces research of greater relevance and utility [7, 8]. Moreover, as those living with dementia (along with carers and families) possess the deepest knowledge of their experience and the greatest direct interest in the research outcomes, it is both ethical and practical to involve them in all stages of research [7, 8, 9, 10, 11].

A public‐involvement approach recognises that individuals with direct experience of health conditions or health service interactions bring perspectives distinct from those of researchers and clinicians who may lack personal patient or carer experience [4, 6, 12, 13, 14, 15, 16]. These perspectives are typically termed ‘lived experience’; however, we propose the terms ‘experience expertise’ and ‘experience expert’, as these terms encompass the unique knowledge of patients, carers and families, while also encompassing past and current ‘lived experience’. ‘Experience expertise’ complements ‘content expertise’—broadly, the knowledge, training and skills typically associated with researchers [16]. Importantly, we do not imply a clear‐cut division between these forms of expertise, nor do we conflate them solely with public partners and researchers, respectively. Indeed, we emphasise that all contributors bring forth diverse and unique perspectives and skills to the process, which complement one another [12, 15, 16, 17].

Research involvement in this sense, conducted ‘with’ or ‘by’ rather than ‘on’ or ‘for’ members of the public, has various labels between countries [9, 18, 19]. In the United Kingdom, it is referred to as Patient and Public Involvement (PPI) [19], Patient Engagement in Research in Canada [20], Patient or Community Engagement in the United States [21] and consumer and community involvement (CCI) in Australia [9]. While these terms all represent a shift from passive consultation to active collaboration, we favour PPI (despite the Australian context), as the term ‘consumer’ for those who engage health services is somewhat contentious [9]. Henceforth, we use PPI throughout this paper. Regardless of terminology, the essential core principle remains to create ‘space’ within the research process in which patients and the public can meaningfully influence conception, design, implementation and thus outcomes of health service research [8]. PPI is not a new concept, particularly in health service research, and is becoming increasingly embedded in research culture [18, 22, 23, 24]. Moreover, it is now a frequent requirement of funding bodies and policymakers [13, 25].

Despite increasing focus, public involvement is not as simple as merely making ‘space at the table’ for public partners [18, 26]. In practice, it is a complex and challenging process that requires concrete support in the form of time, resources and knowledge to facilitate engagement with the public and to include stakeholder needs [4, 9, 13, 18, 22, 26, 27, 28, 29]. Fortunately, numerous resources are available in the literature to guide researchers and public partners in initiating or improving their PPI endeavours [7]. It is advisable to consult information across this spectrum and apply those that best fit one's specific research context. The literature offers many specific examples of PPI efforts where authors detail the actions and considerations used in establishing and operating public advisory bodies [5, 25, 30, 31, 32, 33, 34, 35, 36, 37]. More broadly, there are numerous structured frameworks, guidelines and checklists that can be used to commence, improve or assess PPI efforts [7]. Greenhalgh and colleagues [7] identified 65 frameworks which could be categorised into five groups:

1. Power‐focused: designed to explore researcher‐lay power imbalances
2. Priority‐setting: involving public members in setting research priorities
3. Study‐focused: designed to maximise recruitment and retention for clinical trials
4. Report‐focused: guiding consistent evaluation and reporting
5. Partnership‐focused: designed to assure transparency and accountability in collaborations.

However, there is no ‘one‐size‐fits‐all’ approach [16, 19, 38]. The choice of which examples and frameworks informs one's PPI processes depends on the philosophical approach, research project, group‐of‐interest and level of involvement desired from public partners [13, 16, 39]. The purpose of this Board was to partner with people living with dementia and their carers to co‐design and co‐author specific studies to address the issues that are of the most value to this group. This approach of involving people with living experience of dementia in research in a meaningful way is endorsed by the WHO [8].

The aim of this paper is to describe the development and operation of the eQC Patient and Carer Advisory Board. In this paper, we outline our practical actions and processes during the three phases of Board development outlined by Newman and colleagues: formation, operation and maintenance [40]. By sharing this experience, we hope to provide a comprehensive example to guide other researchers and public partners seeking to establish collaborative research relationships based on a partnership‐focused approach. We will briefly describe successful research and Board operation outputs the Board has partnered in; however, more in‐depth economic, outcomes and experience evaluation of Board activities will be addressed in further papers. This work contributes to the growing body of knowledge of PPI in health research, emphasising its potential to improve health care quality and patient‐centred care.

## 2 Methods

### 2.1 Partnership Across Development Stages

**Figure 1:**
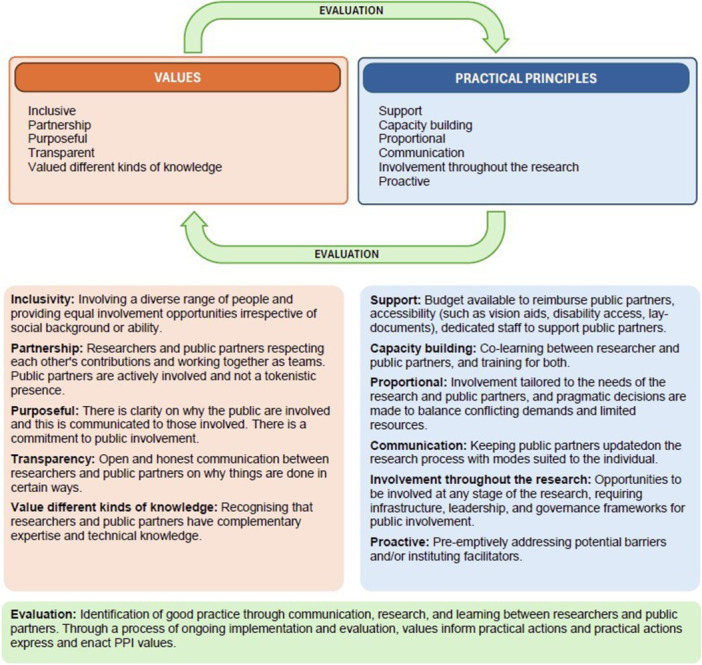
Relationship between involvement values and practicalities, adapted from Liabo et al. [41].

The primary focus of the eQC Project was on older patients', families' and carers' experiences within Australian acute care hospitals. Given this, from among the five framework categories described by Greenhalgh and colleagues [7], a partnership‐focus best aligned with our aims. The Board's development followed three stages: formation, operation and maintenance. During formation, we defined the role and purpose of the Board in relation to the overall project and identified the key experience expertise desired of members. In this stage, we primarily consulted prior examples of advisory bodies and our investigators' (M.M.K. and E.B.) own public involvement experience. As we moved onto the operation stage, we developed our own decision‐making, communication and support processes collaboratively with members. In the maintenance stage—which consisted of evaluation and continuous improvement—[40] we drew guidance from the 11 principles of good PPI (Figure 1) outlined by Liabo and colleagues [41].

### 2.2 Formation Stage

At the eQC Project's inception, the Chief Investigator (CI) (M.M.K.) established the eQC Patient and Carer Advisory Board to contribute experience expertise throughout the research process. The Board's primary roles were to co‐design various subprojects, guide implementation of processes and assist with dissemination strategies for research outcomes.

Beyond the eQC project, the Board's services extended to PhD students, early‐career researchers and other health service research focusing on aged care, dementia and cognitive impairment. This served two key purposes: to create greater opportunity for inclusion of the Board across all stages of research, and to demonstrate the value of patient knowledge to researchers who might not otherwise have resourcing or opportunity to incorporate public expertise.

### 2.3 Member Qualities

Our recruitment strategy aimed to create an inclusive and diverse group of experience experts with broad perspectives of health service utilisation, focusing particularly on people living with dementia or other cognitive impairment, carers (formal/informal), family members and those with intimate knowledge of health service provision (as patients or professionals) (see Box 1). This approach aligned with the values of inclusivity and valuing different kinds of knowledge [41].

Box 1. **Membership. Excerpt from eQC Patient and Carer Advisory Board TOR**

The Board membership includes people who have varied experiences of health service utilisation, and who live in different locations across Australia.

The members collectively bring experience that addresses a demographic of health service users with a particular focus on people with dementia, their care partners and older adults.

The Board membership will collectively have:

- Knowledge and experience of living with dementia or of supporting and caring for a person living with dementia, in the community and/or in formal care.
- Knowledge and experience of living with a chronic condition resulting in extensive health service use;
- Interest in being involved in dementia research and providing input from a lived experience perspective.
- Knowledge and experience of public involvement in research;
- Knowledge and experience of the delivery of health services, and of how to consider proposals for changes to health services delivery.

To ensure robust representation, we prioritised inclusion of at least two members living with dementia or other cognitive impairment, and at least two care partners within an eight‐member group. Furthermore, we aimed to recruit members from different locations across Australia, as well as gender and cultural background to promote diverse and inclusive involvement [41].

### 2.4 Recruitment Process

Our first step in establishing the Board was to appoint an experienced advocate for public involvement in dementia research to the position of Board Chair. We acknowledge that for some advisory bodies, projects and designs, there are legitimate practical reasons for the chairperson role to be filled by an experienced researcher; however, in our context, we opted for a community member to act as chairperson. This decision was driven by several factors, all of which we felt would facilitate a stronger partnership and address inherent power imbalances as much as possible.

- A stronger and more authentic community experience voice in the research
- Greater communication and understanding between researchers and members
- Mutual respect between researchers and members

An ideal candidate for Board Chairperson (J.T.) was already known to the research team (E.B.) and was personally approached for the role. The Chairperson possessed a valuable combination of skills including research experience as a former researcher with a strong interest and past experience in public involvement in dementia research, and experience expertise as a care partner for a person living with dementia.

We sought membership for the Board through newsletters from Dementia Australia and Health Consumers Queensland, along with private and professional networking. The selection process involved unstructured interviews jointly run by the Board Chair (J.T.) and eQC researchers (M.M.K. and E.B.) in which we assessed applicant's suitability for the role and discussed researchers' and the applicants' mutual expectations of what involvement entailed [22]. Co‐led interviews allowed the inclusion of both the researcher, Chair and member perspectives on ‘what membership meant’ and therefore was more inclusive and transparent [41]. Successful applications were jointly decided by the Chair and CI.

### 2.5 Operation Stage TOR

The Terms of Reference (TOR) document (see Appendix A) was developed through a collaborative process between members and eQC researchers. It was drafted initially by the CI, adapted from documents used by similar advisory groups in which the CI and Chairperson had been involved. The draft was reviewed and edited by the Chairperson (J.T.) and a Board member (I.W.) with general member input and was ratified by the Board. The TOR made clear the roles, scope, membership requirements and decision‐making processes. As a jointly penned document, the TOR effort expressed the PPI values of partnership, involvement through the research process, transparency and valuing the different knowledge of eQC researchers and members [41]. The TOR were revised over the period of the Board as required.

### 2.6 Reciprocal Feedback

Two‐way feedback between members and researchers is crucial to effective partnerships [22, 42] and should be communicated in modes suitable to individual member needs [22, 41]. Feedback informs members how and where their experience expertise influences the research process, communicates how their knowledge is valued and facilitates the PPI principle of involvement throughout the stages of research [9, 22, 41, 42, 43].

To ensure meaningful, transparent communication about collaborations, we instituted two key processes. First, after a researcher presented a project for input from the Board, we requested a brief report about their experience guided by the following questions:

- Did you get the kind of input that you were hoping for to assist your project?
- Did the interaction provide you with any unexpected or surprising insights?
- Did your presentation to the Board help confirm decisions you had made for your project (that you are heading in the right direction) or did it help create new directions and ideas for you?

For communicating updates on ongoing collaborations, we established a system to track project stage and present status which recorded next steps and a set date for updates which was either set by the project's researcher or to 6‐months by default. This process kept members regularly informed and promoted transparency regarding progress and decision‐making [41].

## 3 Results

### 3.1 Membership

Appointed Board members sat an initial term of 1 year, with continued membership confirmed via email to the Chairperson and CI. Members could voluntarily terminate their membership at any time with no minimum notice; this pragmatic policy acknowledged that members' circumstances may change at any time. We successfully included two people living with dementia and at least two carers at all times; however, we were less successful in terms of geographic and cultural diversity, with only one member from a culturally and linguistically diverse background, and most members from urban Queensland. In this sense, we were only partially successful in expressing the value of inclusion [41]. Nevertheless, the Board consisted of a cohesive core of six long‐serving members (2020–2024) and a further four who served 1–2‐year appointments (see Table 1).

**Table 1:** Member expertise of the patient and carer advisory board by year.

		Experience expertise			Content/professional expertise		
Year	Members^a^	Dementia/CI	Carer (past/present)	Patient^b^	Research^c^	Public involvement^d^	Clinical experience
2020	8	2	6	4	4	7	4
2021	8	2	6	4	4	7	4
2022	8	3	5	2	3	6	3
2023	7	3	5	2	3	6	2
2024	7	2	5	2	3	6	2

Abbreviation: CI, cognitive impairment.

^a^ Numbers represent the number of sitting members; most members have expertise in more than one category.

^b^ Denotes experience with chronic illness or frequent engagement with health services.

^c^ Denotes experience as a stakeholder representative on research projects.

^d^ Denotes experience with organisations or activities involving public engagement (e.g., Dementia Australia).

^e^ Denotes experience as a health professional.

### 3.2 Maintenance Stage

Rather than a single ‘final’ evaluation, maintenance in PPI is an ongoing process of assessment and improvement of actions and processes [22, 40]. When we encountered barriers, we took note of which PPI values and practicalities were not being sufficiently expressed and attempted to pragmatically incorporate them into our solutions. Board members were key drivers of change, highlighting to researchers when—and often why—processes needed improvement. In PPI, experience and content knowledge are complementary, and this extends beyond the research to include the process. While our approach was partnership‐focused rather than power‐focused, we recognised how power sharing underpins successful partnerships [7, 41]. Highlighting problems ranging from document accessibility to respect was encouraged, as was the collaborative generation of strategies to solve raised issues, thereby facilitating better partnership and respect for different knowledge [41].

### 3.3 Meetings

General Board meetings were held online and commenced in January 2020 and were run bi‐monthly, with the final meeting in November 2024. A 30–60‐min presentation slot was included in each meeting for researchers to present their work and receive feedback. Additional workshop meetings (1–2 annually) were held throughout the project to work on research materials and finalise co‐developed papers.

The use of videoconferencing offered numerous advantages but also presented unexpected challenges. Transmission delays and the loss of body language cues, for example, can lead to accidental interruptions or some speakers unintentionally dominating conversations while others withdraw and spectate [44]. Furthermore, videoconferencing can be cognitively demanding [44], with people living with dementia often more affected by ‘Zoom fatigue’ than others during long meetings [45]. We collaboratively addressed these concerns raised by members and instituted the following changes to the meeting structure:

- 15‐min optional social catch‐up before meeting start.
- 10‐min recess after 50 min.
- Using Zoom's ‘raise hand’ reaction to allow conversation turn‐taking without interrupting.

Furthermore, we co‐produced aid documents consisting of a researcher‐focused guide for running effective online meetings and focus groups (see Appendix B), and a single‐page reference sheet outlining commonly useful Zoom functions for members (see Appendix C). Board members living with dementia highlighted the usefulness of having recordings to revise for memory support.

Consequently, we recorded and distributed recordings postmeeting, which was also of benefit to those unable to attend.

### 3.4 Accessible Documents

Initially, meeting minutes were a record of items and actions; however, Board members highlighted that this format was of limited use due to their infrequent (bi‐monthly) contact with the various projects. In response, eQC researchers expanded the detail in minutes to also record details of the discussion points and provided an overall summary on the minutes first page. To make all documents more accessible, we used 14 pt Arial font, removed acronyms and jargon, used plain language where possible and used physical mail on request, aligning with recommendations by the Alzheimer's Society [46].

### 3.5 Language Use

Board members raised the use of inclusive and positive language and narratives when partnering with the members of the public. Careless language can frame people and their experiences in ways which diminish their value, power, and dignity [4, 22, 47, 48]. Even unintentionally devaluing members' perspectives is not only counterproductive to the goals of public involvement but is disrespectful at best and harmful at worst [22].

Through a series of open and respectful—and ongoing—conversations, we addressed many contentious terms and found more inclusive substitutes (e.g., experience experts and content experts instead of lived experience). The most salient example in our research context, the term ‘dementia’ can be used problematically. Describing a person as ‘suffering’ from or ‘a victim of dementia’, implies diminished capacity and status by placing the condition before the person [48, 49]. Following Dementia Australia's language guidelines [50], we refer throughout this article—and others—to ‘people living with dementia’, without abbreviation or acronym (i.e., PLWD), thereby separating the person from the condition. Constructive language and interactions throughout the research process demonstrate respect for personhood and dignity, adding value to the experiences of individuals in the eyes of researchers, policymakers and the public.

### 3.6 Brief Outcomes

In addition to the current article, Board members have been involved at varying degrees in four published articles thus far [51, 52, 53, 54], two further PPI‐centric articles covering the Board's economic and research impact (currently in draft), and an additional three in the planning stages. The Board also collaborated with eQC researchers to codesign, coauthor and guide dissemination of several dementia‐specific resources for use in acute care hospitals during the pandemic (https://chsr.centre.uq.edu.au/interim-guidance-care-adult-patients-cognitive-impairment-requiring-hospital-care-during-covid-19-pandemic-australia). Board members also provided significant input into a PhD project through feedback on survey items, interview questions and review and proofreading for clarity from the lived experience lens. This PhD was part of the eQC Project and was investigating the implementation of the Comprehensive Care Standard in Australian acute care hospitals [51, 52, 55, 56, 57], for which five articles have been published, with a further three papers under review, and three drafted at the time of writing. Two further PhD projects benefited from the Board's experience expertise: the first focusing on creating quality indicators for Australian Aged Care Assessment Team evaluations, the Board gave feedback on the clarity of results from a carer's perspective for three publications [54, 58, 59]; and the second focusing on developing a picture‐based quality‐of‐life tool designed for use with people living with dementia who may be nonverbal or from culturally and linguistically diverse backgrounds, for which the Board give feedback to refine the pictures before data collection. A full list of publications and outputs from this project can be found here https://chsr.centre.uq.edu.au/patient-and-carer-advisory-board/evaluating-quality-care-project.

## 4 Discussion

The current paper describes the formation, operation and maintenance stages in the establishment of the eQC Patient and Carer Advisory Board, a partnership initiative aimed at enhancing health services for older Australians living with dementia and cognitive impairment. From 2020 to 2024, the Board developed into a cohesive and highly engaged advisory body which built a strong working relationship with the eQC project researchers. We attribute this dynamic to the mutually respectful and collaborative environment informed by a partnership‐focused framework in which we aligned actions and processes with ‘good’ PPI values established in the literature [16, 41, 42]. Our findings are in line with the PPI literature which argues that there is no ideal one‐size‐fits‐all strategy or PPI framework [7, 13, 60]; what functions effectively for one group, project or involvement level will not necessarily produce the same success in others. Flexible adaptation and customisation are key; thus, we do not present this as a ‘definitive’ method. We present this rather as an example of the marriage of process and chosen theoretical framework—partnership—from which other researchers and public partners may draw inspiration for application in their own unique contexts. The following discussion analyses our successes and missed opportunities through the lens of partnership‐focused values and practices, and we offer recommendations for process improvement in similar health service and dementia‐focused PPI contexts and in broader partnered research.

### 4.1 Successful Actions

#### 4.1.1 Chairperson

PPI must be ‘psychologically accessible’—this means that researchers must ensure members feel genuinely included, that they are not ‘talked down to’ (i.e., with complex terminology and jargon) and feel safe to share their perspectives [22, 27]. While the role of Board Chairperson can be (and often is) filled by a researcher, a community member in this context is ideal for a balanced partnership [22]; although, we acknowledge that a public member with research, experience and PPI expertise is hardly the norm [26]. Therefore, should a community chair be desired, capacity‐building should be provided in the areas of research process, group facilitation and PPI if deemed necessary.

We (Board members and eQC researchers) attribute the Chairperson's qualities as being one of the primary facilitators of our successful research partnership. The Chair's combination of research background, experience, expertise and understanding of PPI allowed her to communicate in both ‘languages’—that of members and researchers alike. This enabled her to act in a crucial ‘boundary‐spanning’ role and fostering greater understanding and respect between diverse perspectives [23]. As such, the chair's qualifications helped express perhaps all the values and practicalities outlined by Liabo and colleagues [41].

#### 4.1.2 Membership

Recruiting members presents an opportunity to enact the values of inclusivity, purposefulness and transparency in public involvement [41]. We found loosely structured interviews to be an effective method for assessing potential Board members. Importantly, the interview is about more than assessing experience expertise and other skills relevant to the research context it also allows both parties to clarify mutual expectations of the PPI process and determine how well mutual goals align [22]. In a sense, a casual meeting may be considered the first instance of collaboration, allowing both parties to evaluate the potential for a productive working relationship. While not foolproof, this can help avoid a situation where public members are disappointed by the involvement experience due to misaligned expectations [22]. The interview provides an avenue for both parties to discuss support and capacity‐building needs, helping to avoid a common recruitment pitfall wherein there is a bias towards recruiting public members with preexisting research and communication skills at the expense of more diverse representation [26, 43, 61]. Prioritising needs assessment along with existing skills, a broader range of public perspectives may be included [26]. This should be approached on an individual basis, as needs are likely to differ between people (and indeed change over time) [22]. In our own selection process, the Chairperson and CI conducted the interviews in partnership, which further ensured a Board with a balance of skills and perspectives.

#### 4.1.3 Process Improvement

The aim of PPI is to incorporate a public perspective into health care (and other) research, recognising that members of the public are uniquely placed to recognise barriers and facilitators impacting their quality of care [4, 6, 12, 13, 14, 15, 16]. We discovered this insight also extended beyond research content to everyday processes. Board members often recognised when processes were suboptimal long before researchers did, and their insights were instrumental in developing effective solutions.

In addition to insight regarding process problems, public members often have unique and useful perspectives regarding solutions. Collaborative efforts can be directed towards improving any practical actions which facilitate PPI values, such as capacity building, communication or support [41]. Collaboratively developed processes foster trust and reinforce researcher‐public partnership; thus, such process refinements should be recognised alongside research outcome as significant PPI outputs unto themselves [62].

In our project, process challenges were largely raised informally (i.e., at meetings or via email), which was sufficient due to the strong relationship between Board members and eQC researchers. We did not establish any formal processes from the start, which was a missed opportunity. Relationships take time to develop; therefore, until such relationships are established, formalised avenues for members to raise concerns regarding processes are recommended.

Irrespective of the chosen method, providing such avenues demonstrates commitment to transparency, partnership and valuing members' knowledge [41].

#### 4.1.4 Two‐Way Feedback

Effective feedback from researchers to public collaborators is a critical yet often overlooked aspect in PPI [22]; up to one in five collaborators report being largely uninformed about the influence of their input [42]. Lack of communication can significantly undermine the PPI process.

Feedback to members serves multiple vital functions—it demonstrates that contributions are heard and valued, clarifies which perspectives have influenced researchers' approach and why, and helps contributors learn how to improve their input and boosts confidence for continued engagement [22, 23, 42, 43, 63, 64]. Moreover, given that public partners engage with a project less regularly than researchers, feedback serves to provide essential recaps, supporting recall and maintaining continuity of involvement.

The benefits of a robust two‐way feedback practice extend to researchers as well. Providing feedback prompts researchers to actively reflect on the importance of engaging members of the public, highlights how their insights improve the research approach, and reveals knowledge which may have otherwise been overlooked [22]. Moreover, systematised processes also produce valuable records which document PPI's influence on research practice creating resources for continuous improvement, evaluating success and planning future efforts.

Given the benefits, we recommend implementing two‐way feedback loops between researchers and public partners from the project's inception. Feedback takes time and resources, but through the prioritisation of open and transparent communication on project progress, both researchers and public partners can realise the potential of the research partnership to produce relevant and impactful outcomes.

#### 4.1.5 Language and Stigma Reduction

An initially overlooked but crucial aspect of our PPI process was the language used to speak of and to persons living with dementia and cognitive impairment. While no harm was intended, researchers came to recognise that common, everyday language can implicitly perpetuate negative stereotypes [4, 47]. This can influence even the self‐perceptions of those our project intends to support [4, 48]. The power of language to shape perceptions became clear during interactions between members and researchers.

Reframing a condition through empowering language use during partnership and dissemination of findings can help attenuate stigma and uphold personhood in the eyes of the individual, researchers and readers [4, 48]. Indeed, even in circumstances where language has no meaningful impact on the quality and uptake of findings and recommendations, our overriding goal as researchers in PPI is to do no harm. A respectful and positive experience for public partners is something all researchers should strive for. We therefore recommend that researchers proactively consider their language use. Importantly, we recognise that it is public partners who are best positioned to highlight appropriate language and terminology; thus, we suggest the conversation regarding language use should happen early in the partnership and be revisited regularly.

#### 4.1.6 Online Collaboration

Online collaboration can remove many of the traditional barriers to forming PPI groups; research partnerships are now far less restricted by geography, inaccessible environments, costs and care arrangements for members [9]. However, while solving these issues, online collaboration introduces its own set of challenges [45, 65]. Navigating these challenges requires ongoing dialogue with public partners about their needs and experiences of contributing via online platforms. Regular reassessment and openness to adapt ensures the benefits of online collaboration can be maximised while potential drawbacks are minimised.

#### 4.1.7 Valuing Different Knowledge

A key component of the interaction is a willingness on the part of the researchers to be learners and to be humble in relation to their understanding of PPI. For example, the CI was not experienced at PPI leadership when the grant began, but was willing to ask and be told how to do things better. The implementation of the Board structure and the project outcomes were a result of the collaborative effort. Other researchers and higher degree students who became involved in the project, who may not have understood the full value of PPI prior, soon became strong advocates as they partnered with the Board in co‐designing research.

Public and patient collaborators are not the same as researchers. Learning to communicate to a different audience in written form and in the way you speak is a skill. This does not mean to oversimplify important content and facts; rather, to remove jargon, unnecessary acronyms and not to assume prior knowledge important to comprehension that the reader or listener may not possess. This is, of course, a balance which must be negotiated with one's own public partners.

Communicating research clearly, in detail, and in a way that enables others to provide insight and feedback on gaps is a valuable capacity which can be developed through interaction with public partners.

#### 4.1.8 Missed Opportunities

There were several missed opportunities in our Board formation strategy, which were overlooked during formation or operation and represented process barriers.

#### 4.1.9 Diversity and Representation

Achieving appropriate diversity and representation in PPI is an ongoing challenge in partnered research [9, 61]. Sharma and colleagues highlight a common trade‐off in recruitment: that representativeness in PPI groups often competes with the prioritisation of research/PPI skills [61]. On one hand, these skills are desirable, as skilled public partners are better able to frame their input to fit research priorities; on the other hand, it is argued that these very skills result in public partners who think more like researchers than members of the public [61]. Focusing on the narrow pool of public partners who possess research and public involvement expertise or unconsciously favouring prospective members with better communication skills and those more agreeable to the research process can often exclude more diverse perspectives, resulting in research outcomes which do not adequately represent the community of interest [43, 61].

Despite our intention to recruit a diverse and representative Board, we acknowledge that our efforts fell short of this goal. The Board was majority Caucasian, educated, older than 65 years, female, and located in a single state (Queensland; we had two members interstate [Tasmania, Canberra]).

Critically, we lacked Aboriginal and Torres Strait Islander representation. Reflecting on our approach, we highlight three limitations in our strategy for inviting members: first, we favoured the convenience of personal and professional connections when approaching potential members, including the Chairperson; second, we had limited connections with organisations representing diverse and/or vulnerable groups; and third, we had limited diversity in the research team itself which likely contributed to the first two limitations.

These shortcomings underscore the need for more proactive and inclusive PPI recruitment strategies, particularly as it becomes a more embedded part of health research. Community groups are frequently happy to facilitate public and researcher connections; however, there is often a critical disconnect in communication, resulting in siloed efforts [26]. It is up to researchers who hold positions of power over the research process to do more to develop genuine professional connections with individuals and organisations who may assist in more equitable recruitment. A further consideration for recruitment is retention. In seeking new members for an already well‐established Board, it can be very challenging for an individual person to integrate with that group. This feedback came directly from our members. While we were unsuccessful in later recruitment activities, we attempted to recruit new members in groups or pairs, rather than solitary members.

#### 4.1.10 Capacity Building

Board members identified a lack of training and education as a significant missed opportunity in our foundation and operation process. Training can range from specific skills, which may vary depending on project context to basic understanding of relevant research and clinical practice. Researchers and clinicians take this process knowledge for granted; however, it is not necessarily straightforward to the layperson [26]. Much like feedback, a cursory understanding of processes can help public partners calibrate and tailor their input to be more relevant and effective [26, 43]. Furthermore, capacity building in research increasingly benefits partners with less experience; even short primers and other educational resources can be extremely effective for new members [43]. Previously, we have highlighted the concern of recruiting public partners from a narrow pool of PPI/research‐trained pool [61]; a commitment of effort and resources to capacity building actions for public partners widens the available recruitment pool to include more diverse perspectives [26]. It is therefore important to assess the training needs of public partners early, regularly, and in collaboration with members [22, 43]. Member‐to‐member mentoring is another potential means suggested by the Board, which can increase new member capability and provide meaningful ways for established members to build relationships by passing on what they have learned from their PPI experience.

## 5 Limitations

The primary limitation of this paper is the absence of a comprehensive evaluation of the Board's research impact. Two further publications are in production, which are intended to complement this account of Board development actions. The first aims to evaluate the economic and time costs of formation, operation and maintenance, while the second aims to qualitatively evaluate the impact public perspectives had on researchers and the research process, and which elements of the partnership influenced these perceived impacts. Unfortunately, while our development strategies and recommendations are grounded in established research, we are unable—other than anecdotally—to empirically link our formation, operation and evaluation actions to actual improvements in our partnership. We implemented no methods of measuring baseline and postintervention levels in values (such as partnership, transparency or inclusivity) from process changes. Therefore, while our claims of the presumed effects are backed by PPI evidence, we present only subjective accounts from Board member and researcher experiences. Extending from this first limitation, without objective measures, it is likewise difficult to causally link improved partnership—if any—to impacts on long‐term project outcomes, which may take years to manifest measurably in policy, clinical practice and service improvement. Therefore, this paper in isolation should be viewed primarily as a guide to the integration of a PPI framework's logic and values into practical actions, with a view towards future measurement.

## 6 Recommendations

We recommend grounding PPI actions with a framework—or indeed a combination of frameworks—which align with your own specific research context, involvement level and goals. This ensures that actions and processes are aligned with your objectives. Speaking specifically of partnership‐focused frameworks, ensure that they are embedded from small ‘micro’ actions (like language use and accessible documents), all the way to the ‘macro’ actions (such as research methodology). A comprehensive commitment to partnership from the ground up enables the practical aspects of PPI by aligning them better with partner needs [41].

From our experience, we put forward the following tips for future researchers and public partners alike:

- Read widely, use examples, and become a PPI expert before and throughout your project
- Partner with community leadership
- Have PPI from grant development through to dissemination
- Establish TOR which provide a guide for researchers and public partners alike
- Ensure the status of the public partners is equal to the status of the investigators
- Fund all PPI activities
- Allocate a research staff member (such as an R.A.) to support the board administration
- Embrace continuous improvement throughout your project
- Be humble

## 7 Conclusion

This paper offers practical insights into partnership‐focused strategies for forming and operating a patient advisory body for collaboration with researchers in improving health services. Our experience demonstrates that a focus on partnership and mutual respect fostered a strong relationship between researchers and public partners, which proved instrumental in overcoming process challenges and facilitated collaboration. While there is no one‐size‐fits‐all approach applicable across contexts, there is a growing body of literature which offers numerous examples of PPI groups in various health service contexts, along with evidence‐based frameworks which provide valuable guidance not just on specific actions, but core values and objectives which inform actions. We offer this paper as a guide which highlights the implementation of a framework, with each action in the hope that future PPI efforts can learn and adapt, contributing to the evolving field of partnered research.

## Author Contributions

**Daniel X. Bailey:** conceptualisation, writing – original draft. **Jane Thompson:** conceptualisation, writing – review and editing. **Elizabeth Beattie:** conceptualisation, writing – review and editing, funding acquisition. **Paul Prudon:** conceptualisation, writing – original draft, project administration. **Leanne Jack:** conceptualisation, writing – review and editing. **Jenni Lawson:** conceptualisation, writing – review and editing. **Karyn Lendich:** conceptualisation, writing – review and editing. **Elizabeth Miller:** conceptualisation, writing – review and editing. **Glenys Petrie:** conceptualisation, writing – review and editing. **John Quinn:** conceptualisation, writing – review and editing. **Ivy Webb:** conceptualisation, writing – review and editing. **Leonard C. Gray:** writing – review and editing, funding acquisition. **Melinda Martin‐Khan:** conceptualisation, writing – review and editing, funding acquisition.

## Ethics Statement

2018/HE001582.

## Conflicts of Interest

The authors declare no conflicts of interest.

## Supporting information

Appendix A‐ TOR_February 2022_Final.

Appendix B‐ A4‐Online‐Collaboration_Final.

Appendix C A4‐Zoom‐Quick‐Reference‐Sheet_Final.

## Data Availability

The data that support the findings of this study are available from the corresponding author upon reasonable request.
