# Supplementary material for: Development of a Patient and Carer Advisory Board to Co‐Design Health Services Research for the Quality of Care of People With Dementia
Source: Health Expect. 2026 Apr 5;29(2):e70662. doi: 10.1111/hex.70662 (PMC13051831; doi:10.1111/hex.70662)
Supplement: Supplementary file 1 — Appendix A‐ TOR_February 2022_Final. [file HEX-29-e70662-s002.docx]

The evaluating Quality Care (eQC), led by Chief Investigator Dr. Melinda Martin-Khan, sits within the Quality of Care work at the Centre for Health Services Research (CHSR), The University of Queensland (UQ). The eQC Patient and Carer Advisory Board (The Board) was established in 2020 with funding from the eQC project, a National Health and Medical Research Council (NHMRC) Boosting Dementia Research Project Grant (APP1140459).

1. **Role/Purpose**

The role of The Board is to support engagement of the CHSR with patients and care partners, and to guide opportunities to establish links with the general community.

The purpose of The Board is to inform the embedding of partnerships between lived experience experts and researchers at every level of planning, delivery, monitoring and evaluation of research, translation and policy undertaken by the Quality of Care research group.

The research team, led by Dr. Martin-Khan, values the expert opinions of The Board members, who together will establish strong community collaboration at UQ CHSR for Dr. Martin-Khan’s team, and researchers wishing to work with The Board.

1. **Scope**

The Board will provide advice and feedback to Dr. Martin-Khan and her research team, and the wider research community as appropriate, in relation to current and future research, and policy work.

It will:

- Advise on opportunities to include lived experience perspectives in CHSR research projects;
- Review CHSR project outcomes and advise on relevance to patients and care partners;
- Review research protocols and research materials for readability and ease of participation (including sensitivity to personal issues) for the public;
- Advise on other (non-CHSR) projects or policy work as requested, if appropriate and available;
- Highlight relevant topics of interest that could be the focus of future CHSR research.

Any Board member may represent The Board in specific research or policy work at different stages and in different ways as part of the wider body of work connected to The Board. This may include:

- Reviewing research materials;
- Participating in focus groups and advising on specific topics where nominated;
- Being a member of a project reference or advisory group;
- Being a research partner and/or named investigator on a project;
- Playing an important ambassadorial role for public involvement in research.

1. **Governance Arrangements**

Dr. Martin-Khan’s, is a Senior Research Fellow within the CHSR.

Her research priorities include quality of care and dementia. The Board has been formed as an advisory body to the Quality of Care work carried out by Dr. Martin-Khan, and her team. As such, The Board is not a decision-making authority and is not authorised to make decisions on behalf of the eQC project, CHSR or UQ.

The Quality of Care work includes working with a wide range of collaborators and a large number of universities. The Board is currently funded through an NHMRC Boosting Dementia Research Project Grant held at UQ, but this could change as The Board develops over time.

***Board meeting procedures:***

- Meeting dates are set in advance (preferably by least one month);
- Any member of The Board can nominate agenda items;
- The Board Chair finalises the agenda;
- Matters are discussed amongst the Board members, with due attention to resources and feasibility considerations.

Recommendations from The Board will be forwarded to relevant research teams for implementation decisions by the relevant research team. Research teams will be asked to provide information about project outcomes back to The Board.

1. **Membership**

The Board membership includes people who have varied experiences of health service utilisation, and who live in different locations across Australia.

The members collectively bring experience that addresses a demographic of health service users with a particular focus on people with dementia, their care partners, and older adults.

The Board membership will collectively have:

- Knowledge and experience of living with dementia or, of supporting and caring for a person living with dementia, in the community and/or in formal care;
- Knowledge and experience of living with a chronic condition resulting in extensive health service use;
- Interest in being involved in dementia research and providing input from a lived experience perspective;
- Knowledge and experience of public involvement in research;
- Knowledge and experience of the delivery of health services, and of how to consider proposals for changes to health services delivery.

Board membership will include approximately 10 members at any one time, including a Chair, and in addition to Dr. Martin-Khan as Chief Investigator.

- It is preferable that the membership always includes two people with dementia, and two care partners of a person with dementia (not necessarily together);
- The Chair may delegate the role of Chair to an alternative Board member, research investigator or other person with relevant knowledge and experience;
- Observers may be invited by the Chair or Chief Investigator to attend Board meetings for specific agenda items.

***Appointment Process:***

Vacancies on The Board can be advertised or potential appointees invited personally to apply by the Chief Investigator. All applicants must:

- Complete an application form including the nomination of referees;
- Attend an informal interview either in-person, by video conference or by phone (of 15-30 minutes duration). The interview will be conducted by the Chair, the Chief Investigator and either another Board member or a senior researcher with relevant public involvement in research experience.
- New appointees must be approved by The Board (at a meeting or by email); a quorum of members is required, with a majority in favour for the new Board member to be approved.

1. **Appointment term**

All members are appointed for an initial term of one (1) year. Reappointment occurs by confirmation of involvement after the first year (by email from the Chair or Chief Investigator). The Chair role is held for a maximum of three (3) consecutive years with a Deputy Chair in the final year to be mentored for the role of Chair the following year. The position of Chair can be held more than once.

Any Board member can request a conclusion of membership from The Board at any time via email request to the Chair, copied to the Chief Investigator, with a nominated time frame in which the action is to come into effect. No minimum time for notice is required.

When more than a quorum of The Board retires at the same time, the possibility of staggering retirement dates will be discussed to ensure a smooth transition and if required, at that time there may be more than 10 Board members while incoming and outgoing Board members cross over.

1. **Meeting frequency and location**

Members can attend a Board meeting either in-person or by videoconference. There will be at least three Board meetings a year. Each Board meeting may be two to three hours in duration (or 1.5 hours if by video conference). There will be pre-reading for Board meetings (e.g. draft research protocols, data collection forms, video summary texts, policy statements). Reimbursement of Board meeting time will occur (for a set period).

Members may be asked to read a grant for review (usually a lay summary), taking up to two hours. There may be one grant review per member per year. This could be separate from Board meeting paperwork. Reimbursement of grant review time will occur (for a set period).

Frequency of focus groups depends on research activity. Attendance is voluntary. When location and timing permits, Board members may be able to attend one focus group per year if they wish (either in-person or by remote conferencing). Reimbursement for a focus group will occur (for a set period).

Additional opportunities may be offered to The Board which won’t be reimbursed. These are general communications which have come through the University.

1. **Quorum**

A quorum for each Board meeting will be half the membership plus one (rounded up), not including the Chief Investigator.

A quorum for an extra ordinary meeting will be decided in advance for that meeting and confirmed by the Chair.

1. **Decision making**

The Board will seek to operate on a consensus basis for decision making. Where a consensus cannot be reached on a specific issue, the different opinions will be recorded and the research team asking the question will make a final decision on how to proceed. Feedback on the outcome will be provided to The Board.

1. **Conduct at meetings**

Board members are requested to:

- Regularly attend meetings, actively listen, and engage in relevant and respectful discussion and debate;
- Engage in email communication regarding content either before or after the meeting to finalise meeting matters. Preference is for 100% attendance but in some cases absence is unavoidable.

1. **Management of conflicts of interest and confidentiality**

Members are asked to provide independent and unbiased advice. Declarations of potential conflicts of interest should be shared as they arise.

Members must not share any unpublished material supplied to The Board by the (CHSR team, by people requesting assistance from The Board, or by other Board members, without written permission from the authors. Confidentiality is a priority. If authors are unknown, the information should not be shared.

Personal information that may be shared either during meeting discussions, or via email, verbally or written as a part of Board meetings is also to be treated with confidentiality.

1. **Secretariat**

The team of the connected funded project will provide secretariat support for The Board meetings including:

- Coordinating meeting times and logistics;
- Developing meeting papers;
- Documenting and circulating the outcomes and decisions of meetings.

1. **Resources and support**

The eQC project will provide The Board member(s) with an honorarium payment (based on Health Consumers Queensland rates) for attendance at Board meetings This is intended to cover incidental expenses such as internet and printing costs as well as payment for time and expertise. Other relevant activities will be similarly remunerated.

The eQC project does not cover work on other external projects. When members are involved on such projects, the relevant project will fund their involvement.

1. **Contact details**

For any questions regarding The Board or an EOI please email chsr@uq.edu.au

1. **Review of Terms of Reference**

These Terms of Reference will be reviewed annually or as required.

**Terms of Reference Record**

| **Date** | **Action** |
| --- | --- |
| August 2020 | Terms of Reference drafted |
| February 11, 2022 | Terms of Reference Updated |
