# Supplementary material for: Development of a Patient and Carer Advisory Board to Co‐Design Health Services Research for the Quality of Care of People With Dementia
Source: Health Expect. 2026 Apr 5;29(2):e70662. doi: 10.1111/hex.70662 (PMC13051831; doi:10.1111/hex.70662)
Supplement: Supplementary file 2 — Appendix B‐ A4‐Online‐Collaboration_Final. [file HEX-29-e70662-s001.pdf]

Online Collaboration with Consumers in Research

Consumer and Community Involvement Online

Consumer and Community Involvement (CCI) in research is a collaborative effort between researchers, consumers, and community members who may benefit directly or indirectly from the outcomes of the research efforts. CCI benefits researchers by enhancing study design, improving the validity of findings, increasing community awareness, and highlighting ethical concerns which may not be considered by researchers alone. Consumers benefit from greater alignment of research efforts with community issues, improved translation of evidence into practice, and increased agency in promoting community well-being.

Online meetings using videoconferencing tools such as MS Teams or Zoom are increasingly common tools researchers can use to facilitate wider consumer involvement. Videoconferencing allows participation to be more accessible and convenient than face-to-face meetings by eliminating travel requirements and venue costs. Consumer experts can be gathered from wider geographic locations and can participate from homes or workplaces.

Online meetings introduce complications of their own, however. Barriers include more obvious issues such as consumer computer literacy, disruptions in internet connections, or managing time zone differences. Complications such as 'Zoom fatigue' and difficulties in building group rapport over online platforms can also limit the ability of consumers to contribute as fully as they may in face-to-face meetings.

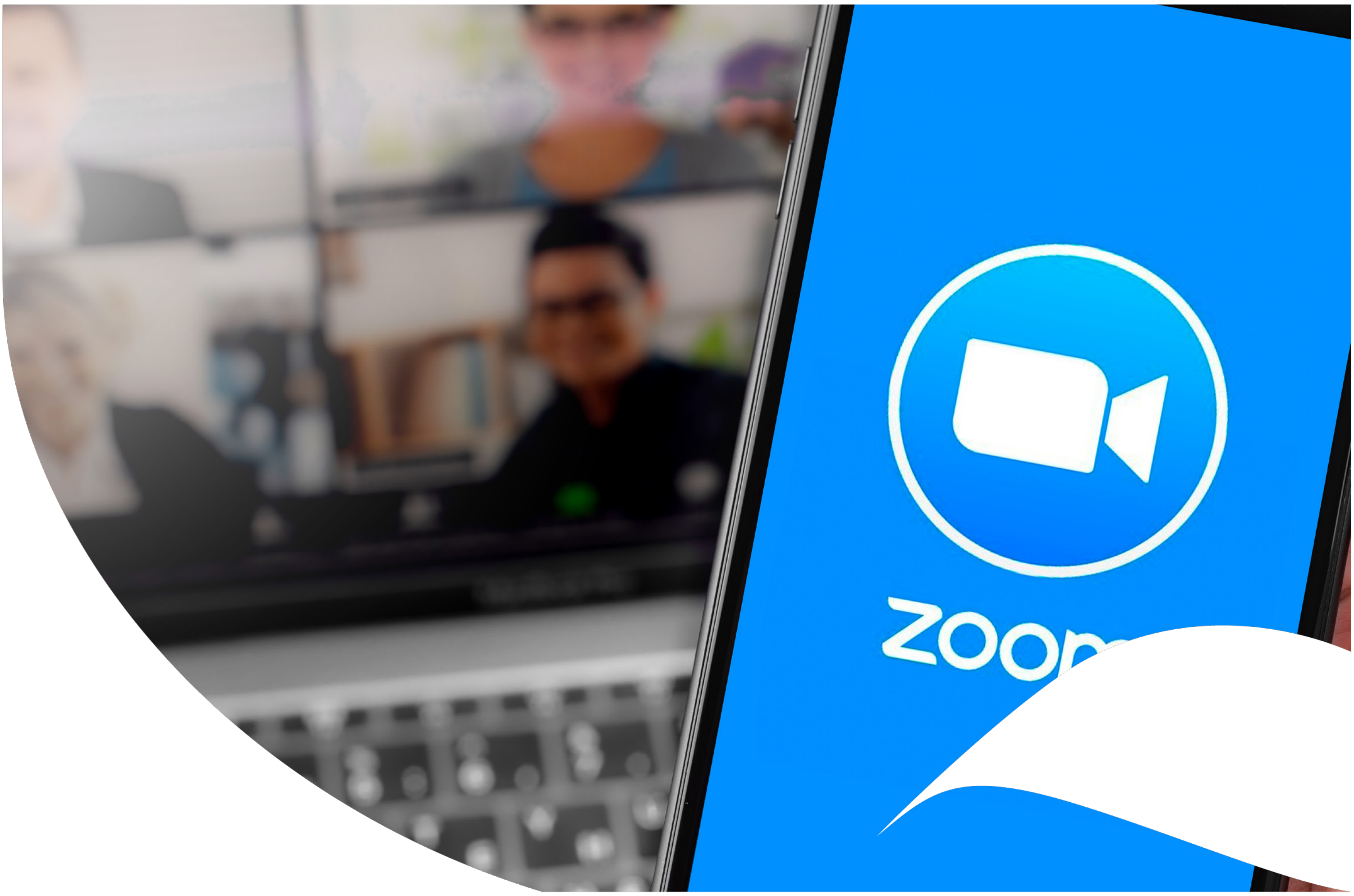

Improving Online CCI Meetings

To improve the CCI experience for your community collaborators, it's important to get their input on specific barriers related to the processes used to contribute to your project. Every research project is different; therefore, every consumer group and their facilitation needs are going to be unique. It is crucial to elicit advice and cooperation from consumer on how to improve their overall experience. The following general recommendations can alleviate some of the more common challenges you and your consumer partners are likely to face when collaborating via online meetings.

Planning

Scaling and Diversity

Scale your consumer group and membership appropriately for the style of contribution your project requires. Smaller groups (3-4 members) allow more 'time-per-member' and thus can provide considerable depth of perspective, whereas a large group (8-12) can provide greater breadth of perspective.

Aid Materials

Ensure you distribute materials required for consumer input with adequate review time. Consider how factors such as font size, formatting, colour, and even digital vs physical documents may impact accessibility of materials. Remember that different consumer groups and individuals have different needs.

Communication

Ensure that meeting dates, agenda, and expectations of consumers are clearly communicated. When emailing information to consumers, ensure that links (to documents or meeting rooms) are easy to locate in the body text, and that the subject line is clear so that emails can be easily searched by consumers.

Time of Day

Consider how time of day affects consumer groups. Older consumers, parents with young children, and full-time carers (to name a few) may prefer meetings earlier in the day, whereas working consumers may prefer meetings outside of business hours.

Running Your Meetings

Start on Time – Finish on Time

Consumers are volunteering their time; therefore, it is important to start and finish meetings on time. Long-standing consumer groups with good relationships may wish to 'catch up' socially at the start of meetings. It is advisable to open the Zoom or Teams meeting 15 minutes early to facilitate socialising without impacting meeting time.

Regular check-in

Don't forget to check in with your consumers regularly during meetings and across the span of the project to elicit feedback on your processes and general social health of the group and be willing to modify processes. Feedback is vital for process improvement.

Breaks

Long periods of on-screen meetings can be cognitively exhausting, sometimes referred to as 'Zoom fatigue.' Some consumer groups such as those living with dementia or other cognitive impairment may find extended meetings fatiguing – ensure you schedule at least one break per hour of discussion.

'Hands Up' or Easing Contribution

In face-to-face conversations, discussion relies upon turn-taking and shifts in participation. Back-and-forth conversation flow via video-conferencing is subtly disrupted by factors such as transmission delays, and difficulty interpreting non-verbal cues such as gaze, facial expression, and body language. Implementing mediated turn-taking (such as using the 'hand up' function in Zoom) can improve conversation flow and ensure all members can find space to participate.

Facilitator Role

In addition to a meeting Chairperson, it can be helpful to appoint a meeting facilitator. The facilitator can monitor text chat, take notes for post-meeting circulation, act as timekeeper, and manage mediated turn-taking. This frees up the meeting Chairperson to manage and fully engage with the discussion.

Meeting Round-up

Documenting the Meeting

Minutes ordinarily highlight key issues which were discussed, decisions made by the group, and assigned actions. However, they are less useful for community collaborators as they provide little information on discussion points. Consider a separate document which provides a richer record of the discussion, or an altered version of minutes which includes this. Work with your group to find the best medium.

Recording

To extract full information, it may be desirable to record your meetings. Ensure that all members consent to the recording and ensure recordings are securely stored. Zoom and MS Teams both have recording capabilities as part of the software. Respect peoples' wishes if they do not wish to be recorded and plan accordingly.

Sourcing Guides for Online Meetings

Communication Matters

There are many guides for using videoconferencing software such as Zoom available on the internet which range in complexity from the very basic up to the very advanced. Talk to your group to find out what their individual needs may be, as solutions may depend on the nature of the community group you are working with. Ultimately, it may be necessary to create your own materials to distribute.

Reference Sheets

A one-page quick reference sheet highlighting the most used commands (such as accessing text chat, cameras, and microphones, etc.) may be helpful. These resources can be kept on-hand by your community collaborators to help them navigate the simple parts of whichever videoconferencing platform you intend to use – any more complicated issues can be referred to and resolved by your meeting facilitator. The eQC Team in collaboration with the eQC Patient and Carer Advisory Board has worked together to create an example Zoom Quick Reference sheet which displays the most used commands for participation in online focus groups. This can be used and adapted as you see fit.

For further details, please contact:

Evaluating Quality Care (eQC) Team
Centre for Health Services Research
The University of Queensland

T: +61 3176 3330
E: chsr@uq.edu.au
W: chsr.centre.edu.au
