# Supplementary material for: Development of a Patient and Carer Advisory Board to Co‐Design Health Services Research for the Quality of Care of People With Dementia
Source: Health Expect. 2026 Apr 5;29(2):e70662. doi: 10.1111/hex.70662 (PMC13051831; doi:10.1111/hex.70662)

Zoom quick reference sheet

The Zoom Toolbar

Basic Zoom functions can be performed using the **Zoom Toolbar** (pictured), which typically appears at the bottom of the Zoom screen. The most useful functions for participating in online Focus Groups have been highlighted.

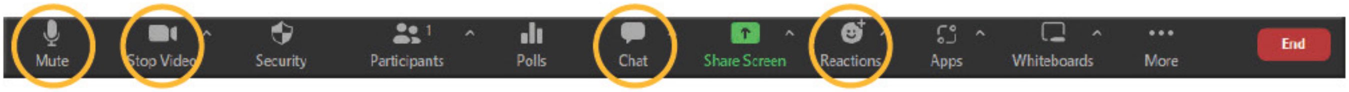

Tablet and phone

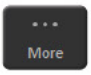

Zoom functions may appear different or be in different places on mobile or tablet versions due to space limitations. If some of the options cannot be seen, they can be accessed through the '**More**' button.

Mute and unmute

- The **Mute** button will toggle your microphone on and off.
- A red slash will appear through the icon when muted.
- You can temporarily unmute your mic by holding the **Space Bar**.

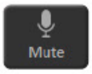

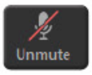

Start and stop video

- The **Video** button will toggle your device's camera on and off.
- As with the **Mute** button, a red slash will appear through the Video icon when your camera is off.

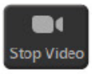

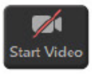

Accessing Chat

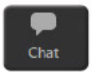

- Press the **Chat** button to open a chat window.
- Type your message and send by pressing the **Enter** key, or the grey arrow on the right of the text entry space.

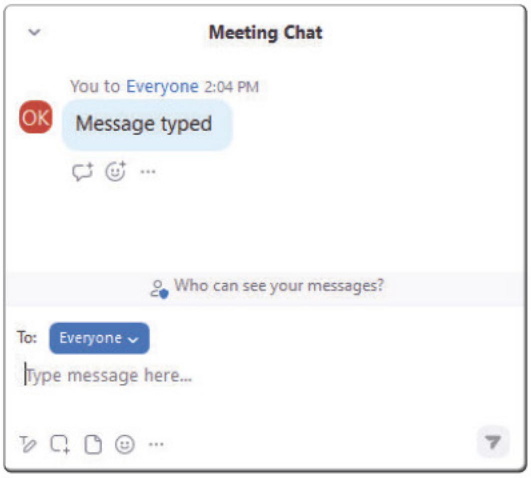

- To chat privately with another meeting member, you can select their name from a drop-down list by pressing the blue **Everyone** button in the Chat window.

Using reactions

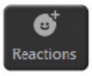

- Click the **Reactions** button to see a range of reactions you can choose from.

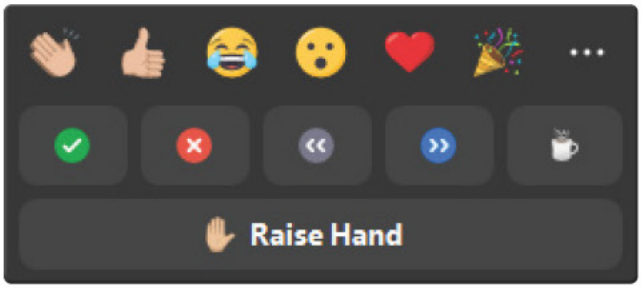

- **Raise Hand** is commonly used in Focus Groups for more orderly discussion, allowing all members opportunity to participate. **Raise Hand** is toggled on and off.
- When your hand is 'raised,' the **Raise Hand** button is replaced with a **Lower Hand** button.
- You can 'lower your hand' by clicking **Lower Hand**.

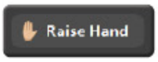

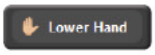

Supplement: Supplementary file 3 — Appendix C A4‐Zoom‐Quick‐Reference‐Sheet_Final. [file HEX-29-e70662-s003.pdf]
